# Race-related differences in the economic, healthcare-access, and psychological impact of COVID-19: personal resources associated with resilience

**DOI:** 10.1186/s41687-022-00514-2

**Published:** 2022-10-17

**Authors:** Carolyn E. Schwartz, Katrina Borowiec, Elijah Biletch, Bruce D. Rapkin

**Affiliations:** 1 DeltaQuest Foundation, Inc., 31 Mitchell Road, Concord, MA 01742 USA; 2 Departments of Medicine and Orthopaedic Surgery, Tufts University Medical School, Boston, MA USA; 3 Department of Measurement, Evaluation, Statistics, & Assessment, Boston College Lynch School of Education and Human Development, Chestnut Hill, MA USA; 4 Department of Molecular Biology and Biochemistry, Middlebury College, Middlebury, VT USA; 5 Department of Epidemiology and Population Health, Albert Einstein College of Medicine, Bronx, NY USA

**Keywords:** COVID, Wellness, Economic, Healthcare access, Psychological, Resilience

## Abstract

**Background:**

The impact of the coronavirus disease 2019 (COVID) is worse among those with fewer financial resources, in jobs not amenable to remote work, and in denser living conditions. People of color are more likely to be among these vulnerable groups. Although race itself is a social construction and not based on underlying genetic/biological differences, this study investigated race/ethnicity differences in the negative repercussions of COVID and in the benefits of psychological and social resources.

**Methods:**

This cross-sectional, web-based study (*n* = 4817) was administered to a heterogeneous United States sample in Spring/Summer 2020. Information was gathered on the following COVID-specific variables: Infection Status, Coping with Lockdown, Social Support, Post-traumatic Growth, Interpersonal Conflict, Worry about Self, Financial Impact on Family, Lack of Money, Inadequate Access to Healthcare, and Housing Instability. Resilience was operationalized as the ability to maintain a sense of wellness in the face of the pandemic, using the DeltaQuest Wellness measure. Multivariate linear regression (adjusting for demographics) and propensity-matched cohort analysis (matched on demographics) evaluated the impact of COVID-specific variables on Wellness in separate models for Whites and Non-Whites.

**Findings:**

Both sets of models retained the same COVID-specific variables and explained about half of the variance in wellness. Coping with Lockdown, Social Support, and Post-traumatic Growth were associated with higher levels of Wellness in both Whites and Non-Whites, while Interpersonal Conflict and Worry about Self were associated with lower levels of Wellness. While these associations are similar, Non-Whites reported worse levels of some positive resources (e.g., social support) and more challenging levels of negative stressors (e.g., interpersonal, worry, financial). Non-Whites also reported much higher levels of post-traumatic growth.

**Conclusion:**

COVID was a source of worry and even conflict, but also unlocked people’s resources in use of health-enhancing behavioral strategies, social support, and renewed gratitude for sources of personal meaning and value. The similar relationships between Whites and Non-Whites on wellness and COVID-specific stressors across racial groups underscore that race is a social construction, not a biological fact. Focusing on a renewed appreciation for sources of personal meaning, and particularly faith, seemed to buffer much of the COVID-related stress for Non-Whites.

**Supplementary Information:**

The online version contains supplementary material available at 10.1186/s41687-022-00514-2﻿.

## Introduction

We are living in extraordinary times. The novel coronavirus disease 2019 (COVID) has led to a world-wide health crisis, infecting over 584 million people, directly causing over 6.4 million deaths worldwide [1], and indirectly causing over 8.5 million excess deaths due to the wider impact of the pandemic on health systems and society [2]. COVID has required enormous changes in our lives. The social distancing needed to contain the pandemic has necessitated shutting down large portions of our economy, with important reverberations for livelihoods [3], healthcare [4–6], healthy living behaviors [7, 8], social connections [9, 10], quality of life [5, 11, 12], and well-being [13, 14]. By dint of its different repercussions associated with sociodemographic [15] and other personal characteristics [16–18], the novel coronavirus also presents a unique opportunity to study social determinants of the impact of such multidimensional challenges.

The COVID pandemic presents stressors at many levels. In addition to the immediate physiological challenges of infection, [19–21], the virus also impacts physical functioning by limiting one’s ability to engage in normal activities, including exercise, if these activities involve being near or around other people [22–24]. The pandemic causes emotional [25, 26] and social stress [27] due to worry [28, 29], hardship [30], social isolation [31], and interpersonal conflict [32] caused either by too much close contact among people with an already stressed relationship, or by difficulty coping with the imposed financial, logistic, or other hardships [33]. The pandemic also causes cognitive stress, as a result of worry, hardship, or other overwhelming situations [34].

In the face of the many negative aspects of the pandemic, what are the most effective ways of remaining resilient to its effects? Psychosocial research across many patient populations points to the importance of social support and social capital [35, 36]. Similarly, conceptual work on resilience in the face of natural disasters has emphasized the “three C’s”: control, coherence, and connectedness [37]. In other words, having a network of people who provide companionship, emotional solace, fun, intellectual stimulation, and pragmatic assistance enables better functioning [38–40]. Additionally, people can attenuate this impact by using coping strategies that are both behavioral (i.e., problem-focused) and cognitive, rather than by focusing on the negative and/or by venting (i.e., negative emotion-focused) [6, 41]. Thus, even when dealing with a stressful and challenging situation, the way people think about it and behave can attenuate or exacerbate its impact [42, 43]. All of these positive behavioral and attitudinal approaches may promote a type of wellness that enables resilience in the face of COVID.

Early in the pandemic, it became clear that the impact of COVID particularly afflicted vulnerable populations, specifically those having fewer financial resources [44–48], being more likely to work in “essential” jobs that were not amenable to remote work accommodations [49], and living in denser conditions [50]. Since people of color are more likely to have fewer financial resources, to work in “essential” jobs, and to live in denser housing, they are at greater risk of experiencing detrimental effects of COVID [51].

It should be noted that race/ethnicity itself is a social construction [52] [53, 54]. It is not based on underlying genetic or biological differences [55], but rather was a construct initiated and promoted for political and financial reasons [56, 57]. With more information about one’s family background comes the recognition that race/ethnicity is likely not a binary personal characteristic, and that many individuals are multi-racial. In the United States population, Hispanic ethnicity is assessed as a variable separate from race. People who are Hispanic in the US are a minoritized group, along with Blacks, Asian, American Indians, and other non-whites. Accordingly, in the present study, we compared Non-Hispanic Whites to all other groups. Although not ideal, this was a way to distinguish “majority” versus “minoritized” participants, and thereby to study social determinants of health and psychosocial resources in the context of the pandemic.

It is unknown, however, whether there are differences in resilience to the pandemic as a function of race/ethnicity. Specifically, is the impact different with regard to the specific negative economic, healthcare-access, and psychosocial repercussions of COVID? Similarly, how do the benefits of psychological and social resources differ by race/ethnicity? The present study addressed resilience using a measure of attitudes, perspectives, and behaviors related to wellness in a large and diverse United States (US) sample.

## Methods

### Design

This cross-sectional study was administered in late Spring through mid-Summer of 2020 (May-15 to July-17 2020), as part of a larger, longitudinal study of the impact of the COVID-19 pandemic on health and well-being. The overall study aims to investigate personal factors related to resilience in response to COVID-related stress in a heterogeneous population in terms of health, race, and ethnicity.

### Sample and procedure

This study recruited participants via Rare Patient Voice and Ipsos Insight —the former to target patients and caregivers of people with chronic medical conditions; the latter to target a general-population sample of US adults who were heterogeneous in terms of health. This general-population subsample was recruited to yield an overall sample that was more diverse and more nationally representative in terms of age distribution, gender, region, and income. Participants were not paid for their participation, although Ipsos Insight used its usual respondent point-related incentives. Eligible participants were age 18 or older and able to complete an online questionnaire. Participants with motor, visual, and/or other problems that made it difficult for them to complete the web-based survey enlisted the assistance of someone else to enter the participant’s answers. This survey was administered through the secure Alchemer engine (www.alchemer.com), which is compliant with the US Health Insurance Portability and Accountability Act. The protocol was reviewed and approved by the New England Independent Review Board (NEIRB #2,021,164), and all participants provided informed consent prior to beginning the survey.

### Measures

*COVID-Specific Questions* included selected items compiled by the US National Institutes of Health (NIH) Office of Behavioral and Social Sciences Research and the NIH Disaster Research program [58]. Additional file1: Table 1 provides a full listing of the items used in the COVID-specific scales and their internal consistency. These items assessed (time frames in parentheses): *Infection Status* (currently) reflecting whether the individual had been infected with Sars Cov-2; *Coping with Lockdown* (currently), reflecting health-enhancing behavioral strategies; *Social Support* (currently), reflecting sources of emotional support; *Post-traumatic Growth* (since the pandemic began), reflecting renewed appreciation for sources of personal meaning and value; *Interpersonal Conflict* (currently), reflecting anger and conflict with others in one’s environment; *Worry about Self* (currently), reflecting dysphoric rumination; *Financial Impact on Family* (currently), reflecting economic problems within the family; *Lack of Money* (past month), reflecting economic concerns of the individual; *Inadequate Access to Healthcare* (during the pandemic), reflecting pandemic-caused lack of access or delays getting medications and routine care; and *Housing Instability* (currently), reflecting not having a regular place to sleep or stay. The Post-traumatic Growth scale used four items adapted with permission from the Post-Traumatic Growth Inventory [59–61].

**Table 1:** Sample Demographic Characteristics (N = 4816)

Variable		White (*n* = 4202)		Black (*n* = 290)		Asian (*n* = 111)		American Indian (*n* = 96)		Hispanic (*n* = 223)		Non-Hispanic (*n* = 4417)		Missing race (*n* = 183)	
		#	%	#	%	#	%	#	%	#	%	#	%	#	%
Role	Patient	2682	64	161	56	31	28	16	17	96	41	2831	64	86	47
	Caregiver	586	14	32	11	6	5	6	6	38	20	602	14	28	15
	Both	163	4	10	3	4	4	1	1	5	3	177	4	3	2
	Neither	704	17	56	19	45	41	6	6	50	27	772	18	18	10
	Missing	67	2	31	11	25	23	67	70	44	19	35	1	48	26
Gender	Male	724	17	41	14	36	32	9	9	38	20	776	18	24	13
	Female	3,392	81	216	74	50	45	20	21	150	79	3,586	82	103	56
	Other	19	0	2	1	0	0	0	0	1	1	20	1	2	1
	Missing	67	2	31	11	25	23	67	70	44	19	35	1	54	30
Living Alone	Yes, living alone	473	11	66	23	14	13	3	3	14	7	549	13	8	4
Marital Status	Never Married	577	14	100	34	29	26	4	4	33	18	690	16	32	17
	Married	2,405	57	68	23	44	40	16	17	106	56	2,446	56	67	37
	Cohabitation	279	7	15	5	3	3	4	4	16	9	294	7	9	5
	Separated	74	2	8	3	2	2	0	0	6	3	82	2	2	1
	Divorced	582	14	50	17	3	3	4	4	22	12	630	14	11	6
	Widowed	208	5	17	6	4	4	0	0	6	3	229	5	3	2
	Missing	77	2	32	11	26	23	68	71	44	19	46	1	59	32
Difficulty Paying Bills	Not at all Difficult	2068	49	79	27	41	37	12	13	76	41	2134	50	52	28
	Slightly Difficult	937	22	59	20	16	14	3	3	39	21	1003	23	29	16
	Moderately Difficult	575	14	55	19	20	18	7	7	28	15	638	15	25	14
	Very Difficult	263	6	30	10	5	5	5	5	20	11	290	7	7	4
	Extremely Difficult	208	5	27	9	3	3	2	2	23	12	221	5	9	5
	Missing	151	4	40	14	26	23	67	70	47	20	131	3	61	33
Employment Status	Employed	1682	40	115	40	40	36	16	17	102	55	1780	41	58	32
	Unemployed	505	12	38	13	14	13	2	2	23	12	547	13	19	10
	Retired	833	20	30	10	15	14	3	3	13	7	870	20	21	11
	Medically Disabled	1061	25	72	25	12	11	7	7	49	26	1123	26	31	17
	Missing	121	3	35	12	30	27	68	71	46	20	97	2	54	30
Education	Less than high school graduate	41	1	3	1	2	2	1	1	4	2	47	1	4	2
	High school diploma/GED	392	9	36	12	5	5	4	4	18	10	412	9	15	8
	Trade or technical degree	267	6	19	7	1	1	3	3	10	5	284	7	8	4
	Some college	1110	26	73	25	19	17	13	14	61	32	1191	27	28	15
	College degree	1226	29	75	26	34	31	3	3	51	27	1302	30	40	22
	Postgraduate degree	1092	26	52	18	24	22	5	5	45	24	1137	26	33	18
	Missing	74	2	32	11	26	23	67	70%	44	19	44	1	55	30
Currently Smoke or Vape	Not at all	3427	82	215	74	74	67	17	18	142	76	3632	83	119	65
	Some days	201	5	17	6	7	6	7	7	19	10	219	5	3	2
	Every day	475	11	26	9	5	5	5	5	25	13	503	12	9	5
	Missing	99	2	32	11	25	23	67	70	47	20	63	1	52	28
Received Help Completing Survey	Yes	65	2	4	1	0	0	2	2	7	4	67	2	3	2
Infected with COVID-19	Yes	302	7	32	11	7	6	6	6	27	14	326	7	14	8

		Mn	SD	Mn	SD	Mn	SD	Mn	SD	Mn	SD	Mn	SD	Mn	SD
Age		52.16	14.07	48.05	13.69	45.63	17.31	47.72	14.47	44.94	12.67	51.98	14.19	51.6	14.19
Body Mass Index		29.9	8.1	31.50	8.6	24.97	4.9	30.890	8.1	30.8	8.7	29.913	8.1	30.0	8.2
Comorbidities		4	2.366	3.57	2.491	2.24	2.55	3.72	2.631	3	2.24	3.670	2.41	4	2.41
Time Since Diagnosis (if applicable)		14.7	13.9	19.71	15.0	19.54	24.3	16.964	14.1	14.0	11.7	15.031	14.2	15.0	14.1

*Some sets of percentages may not add up to 100% due to rounding or because some categories are not mutually exclusive*

*GED General Educational Development (i.e., high-school equivalency test) SD standard deviation*

*The effect sizes are Cramer's v if the variable is categorical and Eta squared if continuous*

*Comparisons by gender across groups exclude "other" gender*

*Mn mean; SD standard deviation*

We conceptualized resilience in the present study as the ability to maintain a sense of wellness in the face of the pandemic. We used the DeltaQuest Wellness Measure© (DQ Wellness), a 15-item measure with documented reliability and cross-sectional reliability, general construct validity, convergent and divergent validity, and known-groups validity [62]. The measure taps attitudes, perspectives, and behaviors relevant to wellness over the past week. Thirteen positively worded items assessed concepts such as joy/zest, self-care/calm, and outward view (i.e., a positive engagement in the world and with others). Two negatively worded items tapped characteristics antithetical to wellness, namely low energy, and a preoccupation with the negative aspects of one’s life. All items followed an instruction to “indicate how true each of the following statements is for you over the past week” and used rating-scale descriptors ranging from “not at all” (0) to “very much” (4). All items provided an option “do not know/prefer not to answer.” The measure yields a general wellness score on an IRT-score metric, ranging from − 3.0 to + 3.0, with a score of zero reflecting the overall population mean.

*Race and ethnicity* were assessed using two questions. The first asked “What is your race? (Check all that apply)”. Eight options were listed: American Indian or Alaskan Native; Middle Eastern; South Asian; Other Asian; Black or African American; Native Hawaiian or Pacific Islander; White; Do not know / prefer not to answer. The second question asked, “What is your ethnicity?” with the following response options: Hispanic or Latino; Not Hispanic or Latino; Prefer not to answer. Following guidelines for coding race/ethnicity used by the National Center for Education Statistics Integrated Postsecondary Education Data System [63], we coded all individuals who reported being Hispanic or Latino as *Hispanic*, regardless of race; and we used multiple codes to reflect all of the races endorsed by each individual.

Other *demographic characteristics* included role (patient, caregiver, both or neither), year of birth (to compute age), gender, with whom the person lives, cohabitation/marital status, difficulty paying bills, employment status, height and weight (to compute body mass index), education, smoking status, total number of comorbidities, year of chronic medical diagnosis (if applicable; to compute time since diagnosis), whether the participant received help to complete the survey, and whether the individual had been infected with Sars Cov-2. Occupational complexity was assessed using the DeltaQuest Reserve-Building (DQRB) measure’s [64] Occupational Complexity Index. Questions querying the job that was closest to the respondent’s current or past occupation were then scored for complexity using the Occupational Information Network (O*NET) system [65]. Under this comprehensive, in-depth job-classification system, scores range from low complexity (1) to high complexity (5)), with higher scores reflecting more training and skills required to perform that occupation [53].

### Statistical analysis

Descriptive statistics summarized the sample demographic characteristics and scores on person-reported outcomes. The COVID-specific variables were evaluated by factor analysis to assess dimensionality of the items within each presumed construct. Alpha reliability coefficients were used to determine whether the internal consistency of the resulting scores were sufficiently reliable for use in multivariate models [66]. Scores were computed as the mean of items with related content multiplied by the number of items in the domain. All COVID-specific variables were transformed to Z scores on the whole sample to facilitate comparison among them and between subsamples. Pearson correlations addressed associations between COVID-specific variables.

Analysis of Variance (ANOVA) models were computed to compare the demographic characteristics of participants across the race groups with at least 26 members (i.e., power to detect at least a large effect size (ES) [67]), and to compare PRO scores by race group (a nominal variable tracking White (1), Black (2), Asian (3), American Indian (4), and Multiple Races (5)). T-tests were used to compare the demographic characteristics and person-reported outcome (PRO) scores of Hispanic versus Non-Hispanic participants. Descriptive statistics were used to illustrate the intersectionality of race and ethnicity, to reflect the multi-racial nature of the study sample, and to summarize the distributions of the PROs used in the present study.

To address selection biases, t-tests were used to compare the demographic characteristics and PRO scores of those missing and not-missing race information. Additionally, we compared the study sample to US census data on gender, age, and US state and region.

Correlation and multivariate linear regression were used to investigate the associations between COVID-specific variables and DQ Wellness. Initial models utilized the whole sample, testing for COVID-specific variables’ effects on DQ Wellness with and without race (i.e., in the second model, dummy variables were included for race/ethnicity groups). Due to small numbers in the different race groups, we had insufficient power to test for interactions with specific race groups in the multivariate models. Additionally, these models suggested collinearity issues and/or suppression due to race/ethnicity differences, so we decided to test for relationships in separate models that stratified by race/ethnicity. For the purpose of these multivariable analyses, the non-White group included Blacks, Asians, American Indians, and Hispanics. Two sets of models were thus computed: one set of models included the *whole sample* and stratified by race/ethnicity (i.e., divided into Whites Only vs. Non-Whites Only); a second set of models focused on the *propensity-matched* samples and stratified by race/ethnicity (i.e., Whites Only vs. Non-Whites Only). The propensity-matched-cohort analysis [68] used the SPSS Propensity Score Matching procedure that includes a FUZZY extension command and aligned the two subsamples (Match Tolerance 0.02) on the following demographic characteristics: age, body mass index, total number of comorbidities, gender, whether the person lived alone, marital status, employment status, occupational complexity, education, smoking status, and time since diagnosis. The FUZZY extension command has several features, including using separate case and control datasets as input, matching on a set of variables without the intermediate logistic regression, and matching multiple controls with each case [68]. The extent of match was assessed by comparing White vs. Non-White group differences on the above covariates using one-way ANOVAs. By having both sets of models, we would thus be able to ascertain what demographic factors were relevant in the Whites Only vs. Non-Whites Only groups, and determine whether matching on these variables modified results.

Both sets of models began with forward stepwise regression selecting among the COVID-specific variables, and then included demographic variables along with the COVID-specific variables. A separate model testing only demographics was included to provide a comparison of explained variance by demographic, COVID-specific, and full models. The following demographic characteristics were considered: age, gender, body mass index, number of comorbidities, educational achievement, occupational complexity, whether the person lived alone, smoking status, time since diagnosis, and Sars Cov-2 infection status.

Due to the relatively large sample sizes and the number of variables examined in the present analyses, we focused on ES of the relevant statistics generated (e.g., beta coefficient (β) for linear models) rather than *p*-value as the criterion for determining relevance of the variables examined. This approach focuses more on the clinical importance, rather than capitalizing on chance significance due to multiple comparisons. We relied on Cohen’s criteria for small, medium, and large ES for interpretation [69]. IBM SPSS version 28 [69] was used for all analyses.

## Results

### Sample

Ninety-four percent of the sample endorsed only one race group, 2% endorsed two race groups, 0.3% endorsed three, 0.1% endorsed 4, and 4% did not provide information on race (data not shown). Table 1 provides sociodemographic characteristics on the sample by race and ethnicity groups, after excluding race categories with fewer than 26 people. Table 2 provides the statistical comparisons of the ethnicity / race groups in the sample.

**Table 2:** Sample Demographic Characteristics (*N* = 4816)

Variable	Differences in Demographic Characteristics for the four groups NOT missing Race/Ethnicity		Differences in Demographic Characteristics by Hispanic Ethnicity		Differences in Demographic Characteristics for the aggregated four groups NOT missing Race vs. those MISSING Race	
	*p*-value	Effect size if sig. (phi/eta2)	*p*-value	Effect size if sig. (phi/eta2)	*p*-value	Effect size if sig. (phi/eta2)
Role	< 0.001	0.129	0.001	0.059	< 0.001	0.506
Gender	< 0.001	0.094	0.423	–	< 0.001	0.173
Living Alone	< 0.001	0.100	0.030	− 0.032	0.029	− 0.032
Marital Status	< 0.001	0.206	0.108	–	0.043	0.049
Difficulty Paying Bills	< 0.001	0.132	< 0.001	0.071	0.333	–
Employment Status	0.002	0.083	< 0.001	0.077	0.644	–
Education	< 0.001	0.111	0.020	0.054	0.258	–
Currently Smoke or Vape	< 0.001	0.083	0.010	0.045	0.066	–
Received Help Completing Survey	0.129	–	0.001	0.050	0.553	–
Infected with COVID-19	0.001	0.062	< 0.001	0.051	0.273	–
Age	< 0.001	0.012	< 0.001	0.498	0.047	0.166
Body Mass Index	< 0.001	0.009	0.293	–	0.590	–
Comorbidities	< 0.001	0.013	0.703	–	0.763	–
Time Since Diagnosis (if applicable)	< 0.001	0.008	0.993	–	0.345	–

*Some sets of percentages may not add up to 100% due to rounding or because some categories are not mutually exclusive,*

*GED General Educational Development (i.e., high-school equivalency test), SD standard deviation*

*Comparisons by gender across groups exclude "other" gender*

*The effect sizes are Cramer's v if the variable is categorical and Eta squared if continuous*

*SD standard deviation*

In the sample, 7% of Whites, 11% of Blacks, 6% of Asians, and 6% of American Indians indicated that they had been infected with Sars Cov-2 (*p* < 0.0001, medium ES; Table 1). There were statistically significant differences by race on all demographics except whether the person received help completing the survey (Table 2). Age and total number of comorbidities were small ES differences. Medium ES differences were detected for Sars Cov-2 infection status, role, gender, difficulty paying bills, employment status, education, and smoking status. Large ES differences were found for living alone and marital status. All others were negligible effect-sizes.

The analyses comparing Hispanics and Non-Hispanics revealed statistically significant differences on all but five demographic characteristics. There were small ES differences on role, living alone, education, smoking status, receiving help to complete the survey, and Sars Cov-2 infection status. There were medium ES differences on difficulty paying bills and employment status. There were large ES differences on age.

#### Selection bias and data quality

**Fig. 1:**
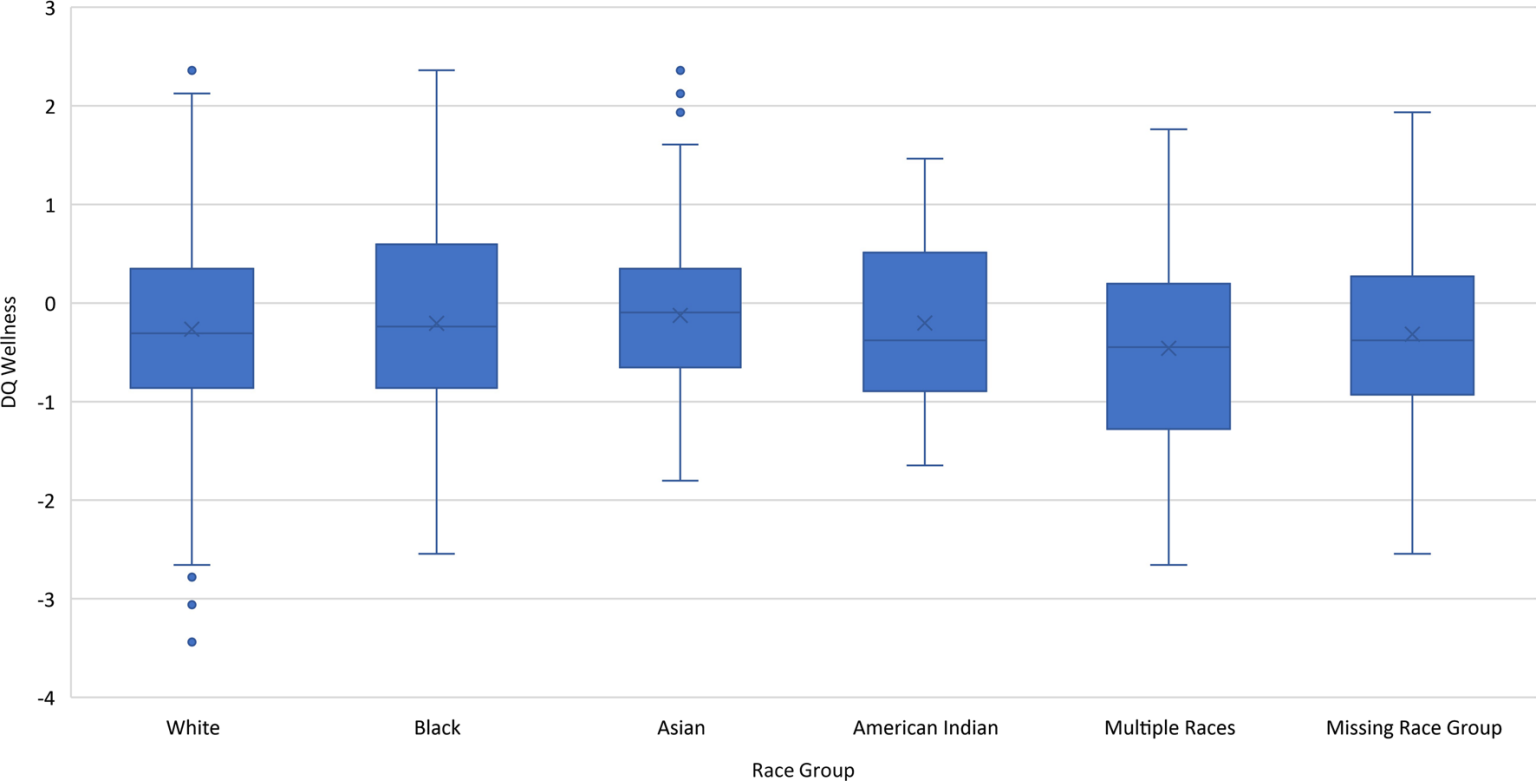
Box and whisker plot showing means and 95% confidence intervals of DQ Wellness scores by race group. Although on average, all participants had negative DQ Wellness scores indicating poor levels of wellness, those with the worst scores were those endorsing multiple races

**Fig. 2:**
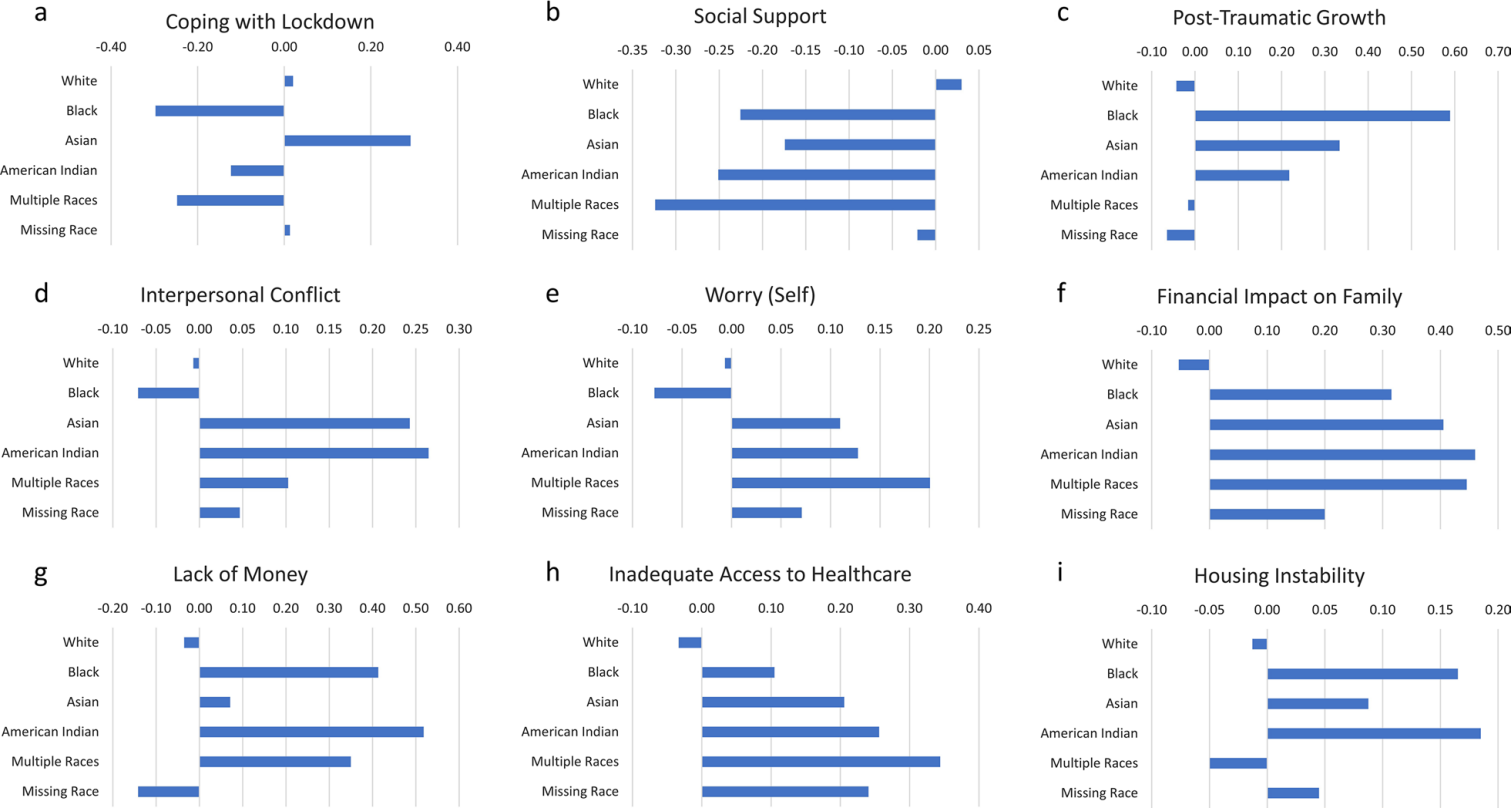
**a**–**i**. Bar chart of racial group differences on COVID-specific variable mean scores. There were marked differences on average levels of all variables compared to Whites

Analyses comparing those participants with and without race information revealed small ES differences in age and gender, and a large ES difference in role (Table 2). Missing race group was associated with being Hispanic although not fully explained by such (Phi = 0.26, *p* < 0.001, data not shown). Comparisons of PRO scores and distributions between Missing vs. Not-Missing race groups generally revealed similar magnitudes of the skewness statistics, although those Not Missing Race had a notably more positively skewed distribution for Financial Impact on Family (Additional file 1: Table 2). Although PRO comparisons by Missing vs. Not-Missing on race group revealed large ES differences on Financial Impact on Family and Inadequate Access to Healthcare (Additional file 1: Table 3), box-and-whisker plots and bar charts revealed that the Missing Race group’s average score tended to be similar to the other Non-White race groups (Figs. 1 and 2 a-i). A comparison between the study sample and US census data revealed that the study sample was 3 years older on average, and included a much larger proportion of women (82% vs. 51%; Additional file 1: Table 4). Although relatively balanced across US regions, the study sample over-represented people in the South, and under-represented people in the West. These selection-bias analyses suggest that the study participants who were missing race data were not notably different from those not missing race information, but they were older, more likely to be female, and over-represented the southern US.

**Table 3:**
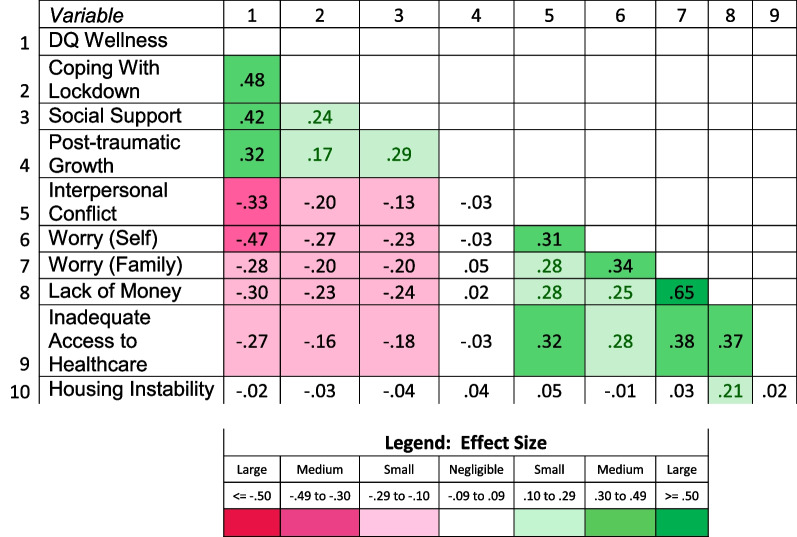
Intercorrelations of DQ Wellness and COVID-Specific Variables

**Table 4:**
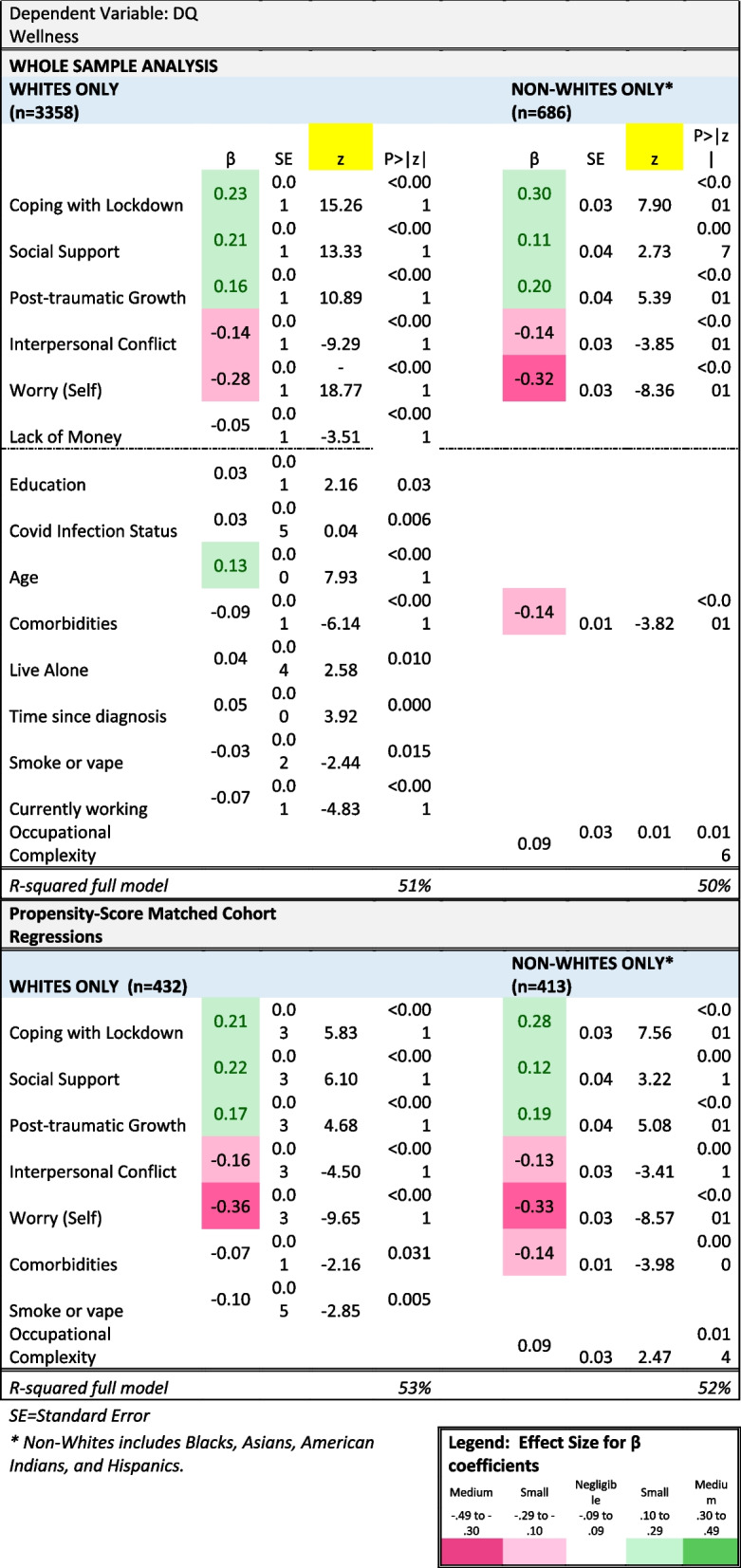
Multivariate Linear Models of COVID-Specific Impact on DQ Wellness by Race Grouping

### COVID-specific variable characteristics

On the COVID-related variables, the most internally consistent scores were Financial Impact on Family, Post-traumatic Growth, Inadequate Access to Health care, and Interpersonal Conflict (α = 0.90, 0.82, 0.77, and 0.76, respectively; Additional file 1: Table 1). Lack of Money, Worry about Self, Social Support, and Coping with Lockdown had somewhat lower internal consistency but were retained in the subsequent analyses because they contained unique information not captured by the other scores (α = 0.67, 0.62, 0.58, and 0.57, respectively).

It was notable that all groups had on average negative wellness scores, meaning that they were below the mean for the norm-referenced sample (Additional file 1: Table 2, Fig. 1). The skewness statistics indicate that while the DQ Wellness score, Post-Traumatic Growth, and Worry about Self were not skewed, several COVID-specific variables had moderate or high skewness in most or all race groups. Coping with Lockdown had a preponderance of high scores among Asians and American Indians (skewness = − 0.56 and − 1.23. respectively). Social support had a preponderance of high scores among Whites and Asians (skewness = − 0.66 and − 0.56, respectively). Interpersonal Conflict, Financial Impact on Family, Lack of Money, Inadequate Access to Healthcare, and Housing Instability had a preponderance of low scores among all groups. Comparisons of Hispanic vs. Non-Hispanic generally revealed similar magnitudes of the skewness statistics, although Non-Hispanics had a notably more positively skewed distribution for Financial Impact on Family and Lack of Money, reflecting a preponderance of low scores on these variables.

#### Intercorrelations

Pearson intercorrelations of DQ Wellness and the COVID-specific variables revealed medium ES correlations with five of the COVID-specific variables, suggesting that higher wellness was associated with higher levels of Coping with Lockdown, Social Support, and Post-traumatic Growth, and lower levels of Interpersonal Conflict and Worry about Self (Table 3). It had small ES, negative correlations with Financial Impact on Family, Lack of Money, and Inadequate Access to Healthcare. It was unrelated to Housing Instability.

The other COVID-specific variables generally had small ES correlations in the expected direction (i.e., psychosocial resources had positive intercorrelations with each other and negative intercorrelations with stress-related variables, and stress-related variables were positively associated with one another; Table 3). Housing Instability was unrelated to all but Lack of Money, and Post-traumatic Growth was unrelated to all of the stress-related variables.

#### Unadjusted group differences

There were no raw differences between race groups in DQ Wellness, Worry about Self, or Housing Instability (Additional file 1: Table 3). Among those COVID-specific variables with significant unadjusted ANOVAs comparing race groups, the eta-squared statistics reflected small ES for Post-Traumatic Growth, Financial Impact on Family, and Lack of Money (eta^2^ at least 0.02). There were large ES differences between Hispanics and Non-Hispanics on Coping with Lockdown, Post-Traumatic Growth, Interpersonal Conflict, Worry about Self, Financial Impact on Family, Lack of Money, Inadequate Access to Healthcare, and Housing Instability (Cohen’s *d* at least 0.80) (Additional file 1: Table 3).

On average, all participants had negative DQ Wellness scores indicating poor levels of wellness, and those reported the worst scores were those endorsing multiple races (Fig. 1). The unadjusted group differences were not, however, statistically significant (Additional file 1: Table 3).

There were marked differences on average levels of all variables comparing Whites and Non-Whites, with statistically significant differences found for all but Worry about Self and Housing Instability (Figs. 2 a-i; Additional file 1: Table 3). In general, Non-Whites reported much lower levels of Social Support, higher levels of Financial Impact on Family, Lack of Money, Inadequate Access to Healthcare, and Housing Instability. In contrast, Asians reported much higher levels of Coping with Lockdown compared to other Non-Whites, and Blacks reported much lower levels of Interpersonal Conflict and Worry about Self compared to Non-Whites. Non-Whites reported much higher levels of Post-traumatic Growth than Whites.

**Fig. 3:**
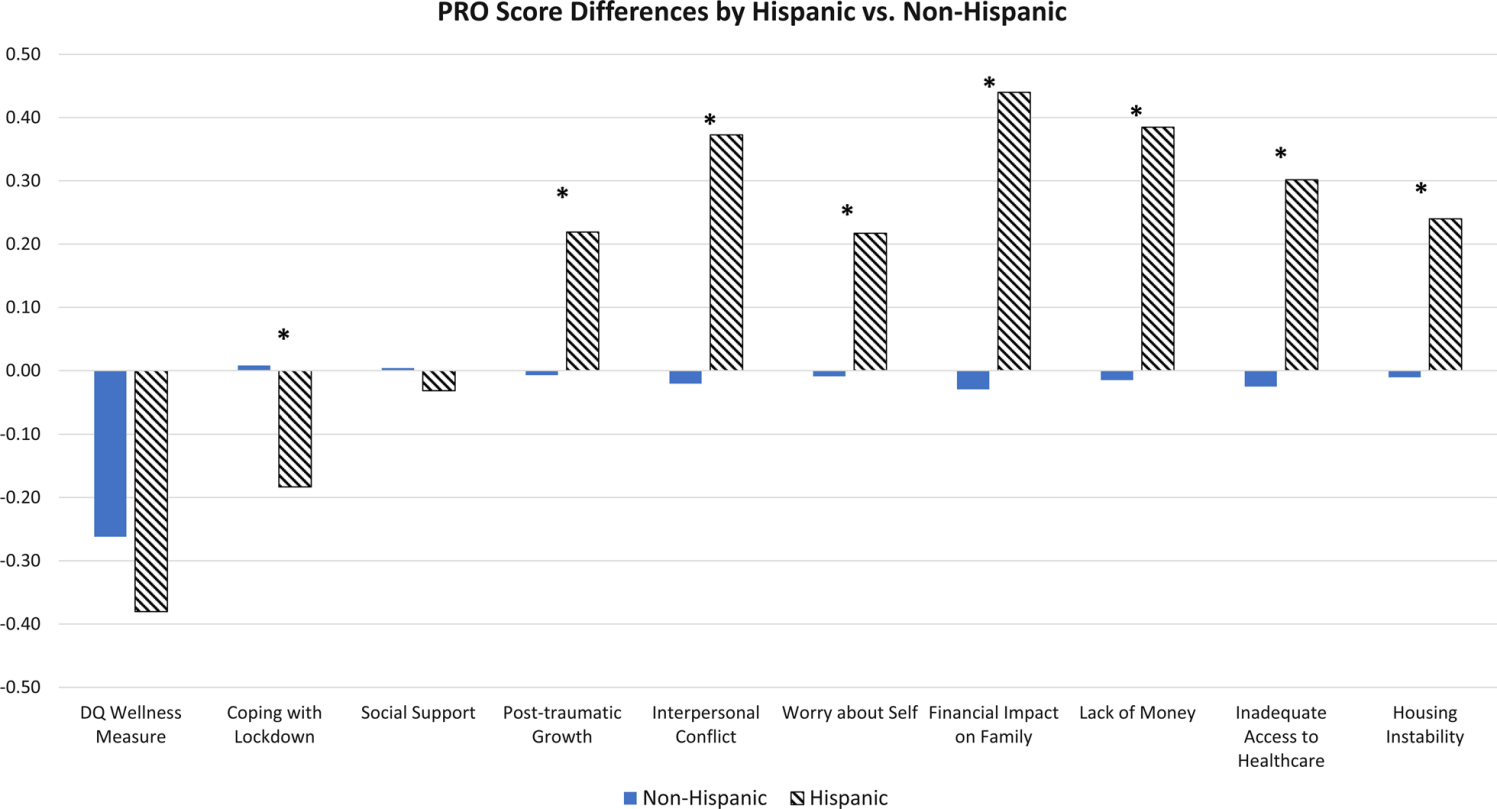
PRO Score Differences by Hispanic vesus Non-Hispanic. Statistically significant differences are indicated by an asterisk above the bars

Hispanics had notably worse scores on Coping with Lockdown, Interpersonal Conflict, Worry about Self, Financial Impact on Family, Lack of Money, Inadequate Access to Healthcare, and Housing Instability (Fig. 3). They had notably better scores on Post-traumatic Growth than Non-Hispanics.

### Multivariate comparisons

The whole-sample full models retained the same COVID-specific variables as the matched-cohort analysis, with generally similar ES magnitude of the beta (β) coefficients. Coping with Lockdown, Social Support, and Post-traumatic Growth were associated with higher levels of wellness in both Whites and Non-Whites, while Interpersonal Conflict and Worry about Self were associated with lower levels of Wellness (top half of Table 4). The Whites-only model also retained Lack of Money, although its ES was negligible (Table 4). A notable difference between the two whole-sample models was what demographic factors were retained. Both retained number of comorbidities, and the Whites had a negligible ES while the Non-Whites had a small ES. The Whites-Only model also retained seven other covariates, for which only age had a non-negligible ES suggesting that older participants reported higher wellness, after adjusting for all other variables. The only covariate retained in the Non-Whites model other than comorbidities was occupational complexity, which had a negligible ES after adjusting for all the other variables in the model.

The propensity matching was effective in aligning the White and Non-White groups on 9 of the 11 demographic and health status characteristics, in contrast to 4 of the 11 in the unmatched subset of the sample (Additional file 1: Table 5). We were able to match 12.7% of Whites and 73.6% of Non-Whites. The lower matching ratio in the White sample is because of the large differences in demographic and health-status characteristics between the White and Non-White samples (Tables 1 and 2). By focusing on a close match (matching tolerance of 0.02), we effectively reduced the sample size and thus power in the propensity-matched analyses. Retaining 73.6% of Non-Whites is justified due to the strict matching tolerance and differences in the demographic and health status characteristics across the groups. We were, however, able to retain a large proportion of the Non-White sample.

**Fig. 4:**
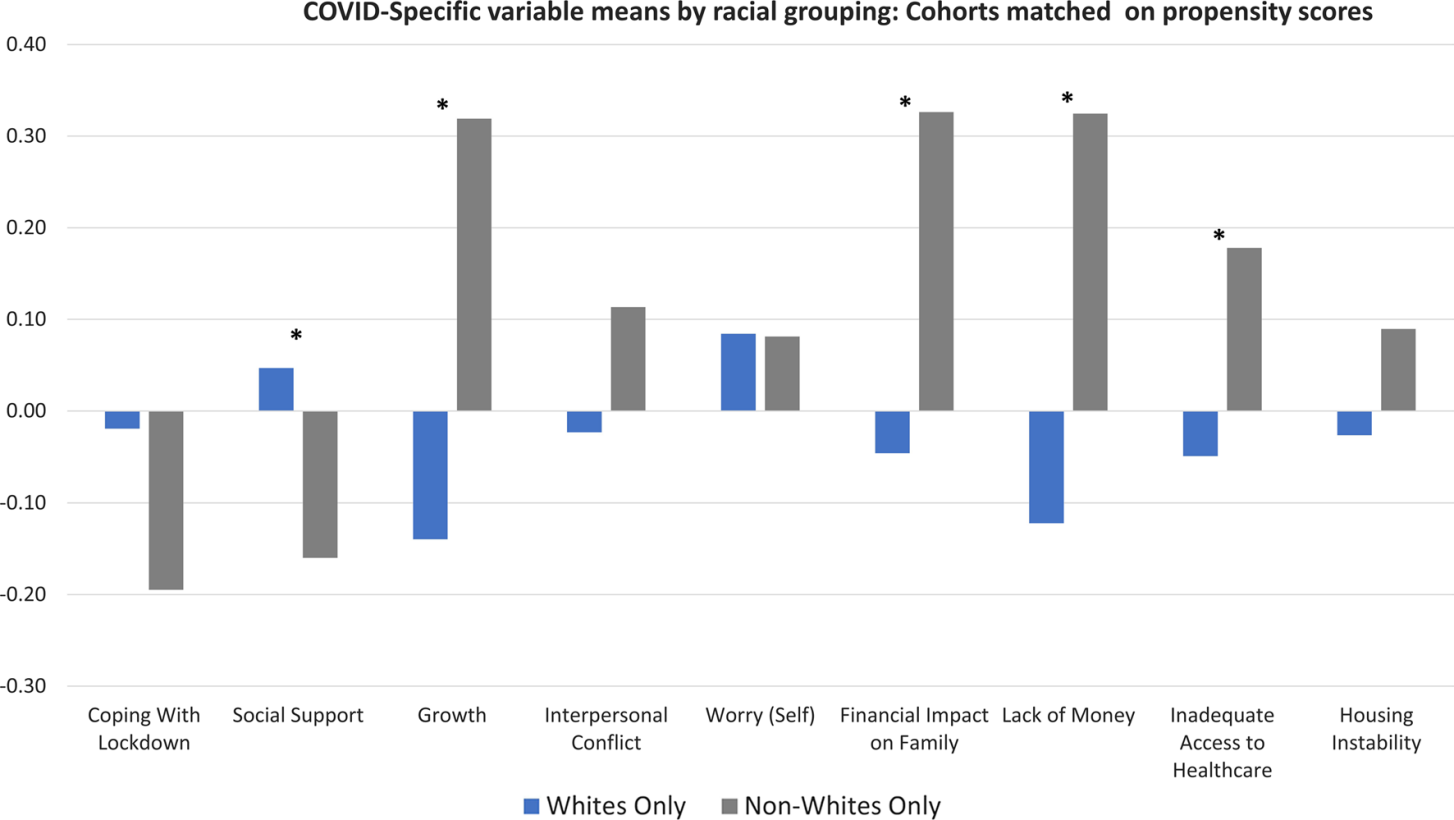
COVID-specific variables by racial grouping: Cohorts matched on propensity scores. Asterisks indicate group comparisons with eta^2^ of at least a small ES (i.e., ≥ 0.01)

Figure 4 shows mean comparisons of COVID-specific variables for Whites vs. Non-Whites in the propensity-matched groups. There were small ES differences in explained variance (eta^2^) for Social Support, Post-traumatic Growth, Financial Impact on Family, Lack of Money, and Inadequate Access to Healthcare. Results of the propensity-matched cohort regressions were very similar to the whole-sample analyses (bottom half of Table 4). The retained COVID-specific variables were identical and had comparable beta coefficients, both in magnitude and direction. The comorbidities variable was retained in both Whites and Non-Whites models, with similar coefficients. Smoking status (for Whites) and Occupational Complexity (for Non-Whites) were retained with negligible ES. Explained variance in the full models was similar albeit slightly higher compared to the whole-sample analysis.

In the whole-sample models, COVID-specific variables explained substantially more variance than demographics, and the full models in Whites and Non-Whites explained about half of the variance (Additional file 1: Table 6). Thus, although mean levels of the COVID-specific variables differed between race groups, their impact on wellness was similar across groups.

## Discussion

Our findings suggest that COVID-specific variables show similar relationships with wellness across racial groups, after adjusting for relevant covariates. Positive resources such as coping with lockdown, social support, and post-traumatic growth, are associated with higher levels of wellness, whereas interpersonal challenges and worry are associated with lower levels of wellness. Even matching the groups on demographic variables in the propensity-matched analysis yielded similar results to the whole-sample, despite the potential for losing power and variability. These similar relationships between COVID-specific stressors and wellness across all race groups underscore that race is a social construction, not a biological fact [52].

While the associations are similar, the average levels of COVID-specific variables differed across race / ethnicity groups. Non-Whites often reported worse levels of some positive resources (e.g., social support) and more challenging levels of negative stressors (e.g., interpersonal conflict, worry about self and family, lack of money, inadequate access to healthcare, and housing instability). These findings are consistent with a large body of research documenting the traumatic effects of racial discrimination and their cumulative impacts [70]. They are also consistent with research documenting that Non-Whites have lesser access to healthcare during COVID-19 [71, 72], and findings of excess mortality rates and premature deaths during COVID-19 for Non-Whites [73]. They are consistent with research documenting that Non-Whites had more difficulty procuring food and supplies during COVID-19 [72]. They jibe with documented evidence that during COVID-19, Non-White women [74] and Black men [75] suffer greater unemployment, and Non-White women experience more substantial losses in their work productivity than men or Whites in general [76]. Our findings underscore the race-related disparities in resources, including social support, healthcare and stable housing.

Our findings suggest, however, a source of resilience for Non-Whites. They reported much higher levels of post-traumatic growth, reflecting their ability to find the good in a difficult situation. Focusing on a renewed appreciation for sources of personal meaning and value, and particularly faith, seemed to buffer much of the COVID-related stress for Non-Whites. It was notable that post-traumatic growth was independent of all of the stress-related variables. In other words, post-traumatic growth co-exists as a distinct dimension from COVID-related stress in all measured domains.

While our study has clear advantages in terms of large sample size, collection of a comprehensive set of informative variables about COVID, and careful modeling, its limitations must be acknowledged. First, the sample had many more Whites than other races, which had the potential to dominate the results. To guard against this, we performed the matched-cohort analyses and confirmed the same findings in smaller, demographically matched samples. Second, although we had a sufficient number of Non-White respondents to compare to the large sample of White respondents, we had insufficient power to test for interactions with specific race groups in the multivariate models. Based on the plots shown in Fig. 2, it is possible that relationships that differed among Non-White race subgroups led to suppression in the multivariable model. Future research might test this study’s hypotheses using large enough racial group samples to be able to test for main effects and race-by-COVID-specific-subscale interactions in a full-sample model. Third, it is not possible to calculate a response rate given the participant-recruitment sources, so the generalizability of the findings is unknown. Nonetheless, the Ipsos comparison sample was specifically recruited to be representative of the adult population in the United States. Further, the selection-bias analyses suggest that the study findings are likely robust to any selection biases caused by missing race/ethnicity information and thus lend support to the generalizability of the findings. Fourth, the COVID-specific measures are based on recommended individual items from the National Institutes of Health rather than scales developed from rigorous psychometric testing. The scales had lower internal-consistency reliability than those commonly used in PRO research. Observed relationships may thus have been attenuated by this imprecision in measurement. Fifth, the models are built from cross-sectional data, and so any causal inference is limited. Future research might assess causality using similar models fashioned from longitudinal data. Finally, the data included in the present work was collected relatively early in the pandemic, and findings might evolve over time. Future work will consider data collected at other time points in the pandemic to address how peoples’ experiences of COVID might change and modify these relationships. For example, did post-traumatic growth peak early and thus have a reduced impact later in the pandemic?

## Conclusion

In summary, although COVID was a source of worry and even conflict, it also unlocked people’s resources in many different ways—use of health-enhancing behavioral strategies, social support, and renewed gratitude for sources of personal meaning and value. Thus, behavioral- and emotion-focused coping clearly contributed to resilience, across all racial and ethnic groups in our sample. The similar relationships between Whites and Non-Whites on wellness and COVID-specific stressors across all race groups underscore that race is a social construction, not a biological fact. Focusing on a renewed appreciation for sources of personal meaning and value, and particularly faith, seemed to buffer much of the COVID-related stress for Non-Whites. Future research is needed to examine how these approaches to coping evolve over the course of the pandemic.

## Supplementary Information

**Additional file 1. Table S1.** COVID-Specific Items and Scales. **Table S2.** Descriptive Statistics of Person-Reported Outcomes. **Table S3.** Unadjusted Group Comparisons on Person-Reported Outcomes. **Table S4.** Representativeness Checks. **Table S5.** Distribution of Covariates in the Matched Samples. **Table S6.** Comparison of Explained Variance by Demographics and COVID-specific Variables in Whole-Sample vs. Matched Cohort Analyses.

## Data Availability

The study data are confidential and thus not able to be shared.

## Abbreviations

COVID: Coronavirus disease 2019
ES: Effect size
PRO: Person-reported outcome
